# A scoping review of breastfeeding peer support models applied in hospital settings

**DOI:** 10.1186/s13006-020-00331-7

**Published:** 2020-11-14

**Authors:** Dorothy Chepkirui, Jacinta Nzinga, Julie Jemutai, Benjamin Tsofa, Caroline Jones, Martha Mwangome

**Affiliations:** 1 Centre for Geographic Medicine (Coast), Kenya Medical Research Institute/Wellcome Trust Research Programme, P.O. Box 230, Kilifi, 80108 Kenya; 2 Department of Public Health, School of Human and Health Sciences, Pwani University, Kilifi, Kenya; 3 Centre for Tropical Medicine and Global health, Nuffield Department of Medicine Research Building, University of Oxford, Old Road Campus, Roosevelt Drive, Oxford, OX3 7FZ UK

**Keywords:** Breastfeeding peer supporters (BFPS), Breastfeeding, Peer support

## Abstract

**Background:**

The 2013 updated guidelines on management of severe acute malnutrition in infants and children recommends the support of exclusive breastfeeding. These guidelines are inconsistently applied in low and middle income countries (LMICs) due to barriers including unclear implementation guides, technical support and epidemiological factors. Peer support strategies have been used to offer psychological support to families with infants in NICU and improve mental health outcomes. Breastfeeding peer supporters (BFPS) have been shown to be effective in improving breastfeeding outcomes in community settings however, their success within hospital settings in LMICs is unknown. We conducted a scoping review to explore implementation of breastfeeding peer support strategies as have been applied to hospitalized infants globally and highlight their implementation strategies in order to guide future research and practice.

**Methods:**

A scoping review of the literature was conducted using the Arksey and O’Malley framework. A search was conducted in five online databases (PubMed, Cochrane library, Hinari, Google Scholar and Open Grey library). Data were extracted and charted in data extraction tables to capture general characteristics, modes of peer support delivery, implementation details and evaluation procedures.

**Results:**

From the online search 276 articles were identified, however only 18 met the inclusion criteria for the study. The majority of these articles were reports on in-patient breastfeeding peer support interventions applied in Europe and the United States of America and only two were from LMICs. The articles described peer supporters’ identification, training (*n* = 13) and supervision (*n* = 14). The majority of the BFPS were employed (*n* = 10) compared to volunteers (*n* = 3) and support was mainly one-to-one (*n* = 11) rather than group support. Process and impact evaluation (*n* = 13) reported positive breastfeeding outcomes associated with breastfeeding peer support.

**Conclusion:**

Breastfeeding peer support strategies are applied in different hospital settings and can be used to improve breastfeeding outcomes. However, to achieve integration, scalability and comparability of impact and outcomes, there is a need to standardize training, develop consistent implementation and supervision plans of in-patient peer supporters’ strategies. Further research to assess sustainability and evaluate cost-effectiveness of in-patient breastfeeding peer support strategies will improve uptake and scalability of these potentially lifesaving interventions.

## Background

In 2013 the World Health Organization (WHO) updated the treatment guidelines for malnourished children and for the first time included a section on how to identify and manage severe acute malnutrition (SAM) occurring in infants under six months of age (u6m) [1]. The cornerstone of the recommendation for u6m is the establishment or re-establishment of exclusive breastfeeding (EBF) with discharge only when weight gain is observed on breast milk alone for three consecutive days [1]. This is in line with the promotion, support and protection of breastfeeding in the initial declaration of Innocenti of 1990 [2]. However, while the guidelines provide a strong recommendation on establishing or re-establishing breastfeeding during hospitalization, the implementation strategies for this recommendation are not clearly defined. There is limited description on how to optimize and support breastfeeding in an inpatient setting. Evidence from three low and middle income countries (LMICs) indicate low adoption and implementation of the WHO 2013 guidelines even at the community level due to technical, political, operational and epidemiological barriers [3].

The use of peer support to identify and manage different health conditions is not a new concept. Peer supporters have been shown to provide psychological and mental health support to families in neonatal intensive care units (NICU) [4, 5], to those experiencing mental health conditions [6] and to people living with HIV [7]. Peer support has been recognized for its high reach to minority and hard to reach groups and fills the role of humanizing healthcare [8]. Breastfeeding peer supporters have been used successfully to support breastfeeding mothers in community settings [9, 10]. For example, in Kenya, two studies have reported using breastfeeding peer supporters to increase the rate of exclusive breastfeeding in a poor urban community setting [11, 12]. However, little is known about their ability to support mothers of ill infants admitted within hospital settings.

A recent study in Kenya explored the use of BFPS in a hospital setting to support ill malnourished infants aged between one and four months [13]. Findings from the study indicate that BFPS are effective to increase the rate of exclusive breastfeeding in hospitalized infants. Qualitative data from this study indicates that BFPS work by creating an emotional bond with the mothers during the admission period. This helps to generate trust giving confidence in mothers to explore new ideas and techniques as suggested by the BFPS [14, 15]. In this way BFPS can provide the more intensive breastfeeding support which is recommended for infants admitted with an illness who are likely to experience low appetite and have little energy to breastfeed by themselves. We set out to conduct a scoping literature review to explore implementation of breastfeeding peer support strategies that have been applied for hospitalized infants globally and highlight similarities and differences in their implementation in order to guide future research and practice.

## Methods

Previous experience had indicated a scarcity of literature on breastfeeding peer support models applied in hospitals within low income countries. A scoping review study design was undertaken to identify the types and quantity of information available on hospital based breastfeeding peer support strategies. We applied the Arksey and O’Marley framework [16] for conducting scoping reviews. We followed the six steps for the scoping studies framework and selected studies that met the inclusion criteria. In stage one and two we defined the research objectives, search terms and conducted a literature search in five online databases (PubMed, Cochrane library, Hinari, Google Scholar and Open Grey library). Table 1 indicates search terms and search details used for PubMed database. The search included the terms peer support, breastfeeding and hospital-based support. The search was widened by the identification of relevant search terms and their synonyms. The online data search was concluded in November 2018. We sought for additional articles by searching through bibliographic citations. In the third stage, we selected relevant articles based on inclusion and exclusion criteria.

**Table 1:** Article search on PubMed

	Search terms	Search details
1	Breastfeeding peer supporters AND hospital	((“breast feeding”[MeSH Terms] OR (“breast”[All Fields] AND “feeding”[All Fields]) OR “breast feeding”[All Fields] OR “breastfeeding”[All Fields]) AND peer [All Fields] AND supporters [All Fields]) AND (“hospitals”[MeSH Terms] OR “hospitals”[All Fields] OR “hospital”[All Fields])
2	breastfeeding support AND inpatient	((“breast feeding”[MeSH Terms] OR (“breast”[All Fields] AND “feeding”[All Fields]) OR “breast feeding”[All Fields] OR “breastfeeding”[All Fields]) AND support [All Fields]) AND (“inpatients”[MeSH Terms] OR “inpatients”[All Fields] OR “inpatient”[All Fields])
3	hospital based breastfeeding peer supporters	(“hospitals”[MeSH Terms] OR “hospitals”[All Fields] OR “hospital”[All Fields]) AND based [All Fields] AND (“breast feeding”[MeSH Terms] OR (“breast”[All Fields] AND “feeding”[All Fields]) OR “breast feeding”[All Fields] OR “breastfeeding”[All Fields]) AND peer [All Fields] AND supporters [All Fields]
4	hospital based breastfeeding peer support	(“hospitals”[MeSH Terms] OR “hospitals”[All Fields] OR “hospital”[All Fields]) AND based [All Fields] AND (“breast feeding”[MeSH Terms] OR (“breast”[All Fields] AND “feeding”[All Fields]) OR “breast feeding”[All Fields] OR “breastfeeding”[All Fields]) AND peer [All Fields] AND support [All Fields]
5	peer counselors AND breastfeeding AND hospital	(peer [All Fields] AND (“counsellors”[All Fields] OR “counselors”[MeSH Terms] OR “counselors”[All Fields] OR “counseling”[MeSH Terms] OR “counseling”[All Fields])) AND (“breast feeding”[MeSH Terms] OR (“breast”[All Fields] AND “feeding”[All Fields]) OR “breast feeding”[All Fields] OR “breastfeeding”[All Fields]) AND (“hospitals”[MeSH Terms] OR “hospitals”[All Fields] OR “hospital”[All Fields])
6	Breastfeeding AND mentor mothers OR counselors AND hospital	((((((((“Breast Feeding”[Mesh] OR “Milk, Human”[Mesh]) AND “Peer Group”[Mesh]) OR “Mentors/psychology”[Majr]) OR “Mothers”[Mesh]) AND “Social Support”[Mesh]) OR “Counselors”[Mesh]) OR “Counseling”[Mesh]) AND “Hospitals”[Mesh])

### Study selection criteria

We included articles that described the use of lay breastfeeding peer supporters in hospital settings. Lay BFPS are mothers who are literate but have no college training or formal health work experience. Articles indicated any type of peer support regardless of the term used, for example, mother to mother support, lactation counsellors. Articles were either from peer reviewed journal publications or grey literature. The articles reported support offered to mothers of infants u6m, and not limited to any geographic region or year of publication. This was to increase the range of articles identified for this review.

We excluded articles that did not have a clear indication of a model of breastfeeding support; those that described breastfeeding support offered at an outpatient clinic or at community levels and those that recruited participants in the hospital but offered breastfeeding support outside the hospital.

### Data extraction and analysis

Two reviewers were involved in the selection of the studies and independently participated in data extraction and charting. Two templates were developed and used to capture the data. The first template included the general study characteristics while the second template contained information on implementation details and evaluation processes. Extraction of data was done continuously until all relevant information to answer the research question had been identified. After charting was completed, the type and distribution of the articles were organised according to geographic region and year of study or publication of the article. Graphs were used to give a simple description of the articles. A summary of the findings was organized to describe similarities and differences in the studies, and depth of the vast knowledge obtained.

## Results

### Search outcomes

**Fig. 1:**
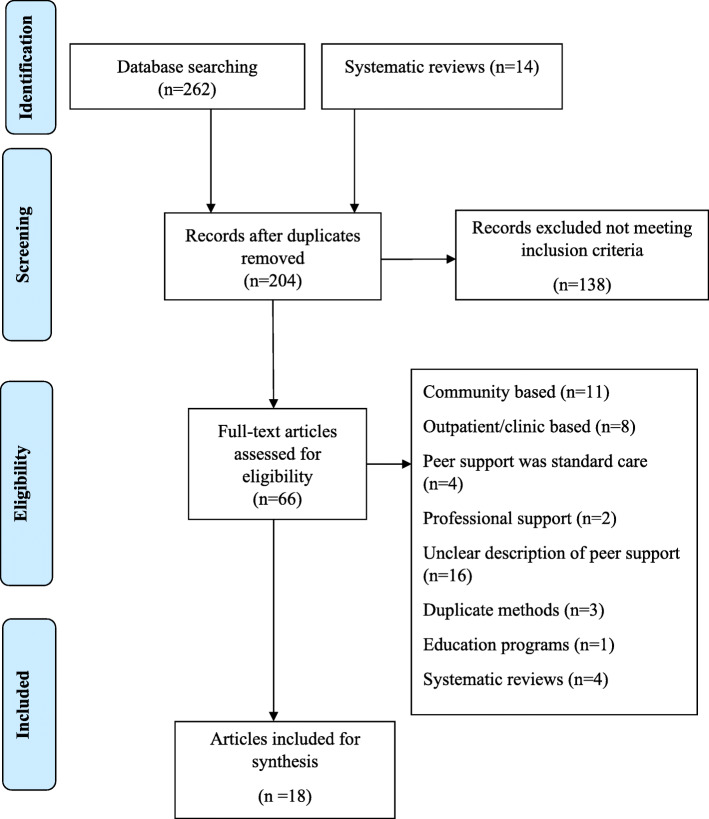
Search selection process

A total of 276 articles were identified from which 95 duplicates were discarded. The remaining 181 articles were screened by title and abstract while applying the inclusion criteria where 138 articles were excluded for being irrelevant to the review question. Full texts of the remaining 43 articles and an addition of 23 articles from biographies were reviewed and further subjected to inclusion and exclusion criteria. A total of 18 articles met the inclusion criteria and were included in the final synthesis of findings. The majority of the studies excluded described breastfeeding peer support in a community setting. Other reasons for exclusion included unclear description of the peer support program, outpatient breastfeeding peer support, breastfeeding peer support offered was standard care and professional breastfeeding support. Figure 1 is a flow diagram indicating the selection process for papers included in the final synthesis.

### Characteristics of the studies

**Fig. 2:**
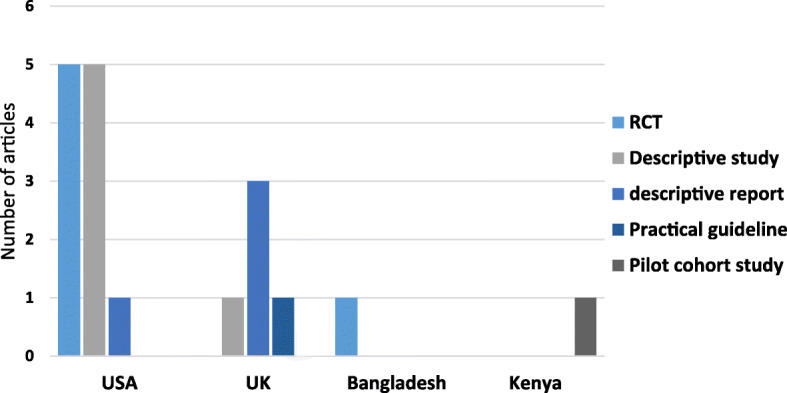
Characteristics of the articles per geographic location and study design

The articles retrieved and included in this review varied greatly in the study design and geographic setting in which the studies were conducted (Table 2). Most of the studies were undertaken in high income countries (*n* = 16) while only two were from low and middle-income countries (LMICs). Studies took place in the United States of America (*n* = 11), the United Kingdom (*n* = 5), Bangladesh (*n* = 1) and Kenya (*n* = 1). Seven of the papers were simple descriptive reports, six contained reports of randomized controlled trials, one pilot cohort study, and a practical guideline. The distribution of the study per country and study design is shown on Fig. 2.

**Table 2:** Studies included in the synthesis

	Author (year)	Country	Hospital setting	Type of peer support	Duration of peer support	Training for peer supporters
1	Anderson, et al. (2005) [17]	USA	Postpartum ward	Contracted during the study period offering One-to-one support	Daily during hospitalization then post-discharge up to 6 weeks postpartum	Done using the WHO/UNICEF training module
2	Chapman, et al. (2013) [18]	USA	Prenatal/Postpartum and inpatient	Contracted during the study period offering One-to-one support	Daily during hospitalization then post-discharge up to 6 months postpartum	30 h of classroom training and 3 to 6 months close follow up
3	Chapman, et al. (2004) [19]	USA	Postpartum ward	Contracted during the study period offering One-to-one support	Daily during hospitalization then post-discharge up to 3 months postpartum	“Topics covered include breast anatomy and physiology, management of breastfeeding, counseling techniques, and related cultural and social factors”
4	Merewood, et.al (2006) [20]	USA	Neonatal Intensive Care Unit	Contracted during the study period offering One-to-one support	In hospital for 6 weeks done weekly for at least 30 min	5 day training by The Center for Breastfeeding, NICU procedures and mandatory regular training.
5	Haider, et al. (1997) [21]	Bangladesh	Paediatric unit	Contracted during the study period offering One-to-one support	3 counselling sessions before discharge first lasting for 5 to 7 min then the other two 30 to 40 min	3 week training, using the breastfeeding counselling course for health workers
6	Oza-Frank, et al. (2014) [22]	USA	Neonatal Intensive Care Unit	Employed by the national hospital Unclear the type of peer support	During hospitalization only	Physiology of lactation, infant medical conditions, and the benefits of breastfeeding.
7	Kristoff, et al. (2014) [23]	USA	Neonatal Intensive Care Unit	Volunteers giving own experiences in a Mother-to-mother Group support	While hospitalized done once a month	No training offered
8	Meier, et al. (2013) [24]	USA	Neonatal Intensive Care Unit	Employed peer supporters by the hospital to offer Combined one-to-one and group support	While hospitalized Peer support available 14 h weekdays and 8 to 9 h weekends	Trained through La Leche League International
9	Ahluwalia, et al. (2000) [25]	USA	Postpartum ward	Model not clearly described	Not described	Not described
10	Merewood, et al. (2003) [26]	USA	Postpartum ward, NICU, telephone model	Employed Peer supporters by the hospital through small grants offering three types of support 1. Telephone support 2. One-to-one support postpartum unit 3. one to one support in NICU	while hospitalized Unclear for telephone model in the postpartum model peers available 4 days a week for 4 h	Trained using Massachusetts State WIC peer counselor manual for 1998 and counselling skills
11	Hooper, et al. (2016) [27]	England	Postpartum ward	Volunteer Peer supporters giving One-to-one support	While hospitalized	10 weekly 2 h training organized by the community health care trust (UNICEF BFHI)
12	Pugh, et al. (2002) [28]	USA	Postpartum ward	One to one support done through combined Peer counselor and a community nurse. Both are employed by the hospital	Daily during hospitalization up to 6 months postpartum	Yes, but training details not described
13	Devon Integrated Children Services, (2012) [29]	UK	–	Done by either employed or volunteers using a one-to-one support or group support mode	–	Should be done by an accredited organization e.g. La Leche League and should include an assessment of participant knowledge and awards given.
14	Singleton, (2018) [30]	USA	Postpartum ward	Employed by the county through partnership offering one-to-one support	While hospitalized	Not described
15	Potter, (2013) [31]	England	–	Volunteers but unclear on the type of support	–	10 weeks of training by an accredited training program
16	Healey, (2013) [32]	UK	Maternity, neonatal and paediatric unit	Employed peer by Wigan public health offering one-to-one support	While hospitalized	Not described
17	Whitmore, (2013) [33]	England	Maternity, neonatal and paediatric unit	Combined employed and volunteers by Blackpoll council through small grants to offer one-to-one support	While hospitalized	Unclear
18	Mwangome, et al. 2019 [13]	Kenya	Paediatric ward	Contracted during the study period offering one-to-one support	Daily during hospitalization up to 6 weeks post-discharge	Yes. 5-day training on introduction to lactation management. (UNICEF, BFCI, WHO etc.)

The target populations included in each study differed based on the condition of the child and the mother. Four studies [13–15, 28] described breastfeeding peer support offered to the mother who had interest in initiating breastfeeding even though their infants had no clinical illness. These were categorized as healthy infants. Seven studies [13, 15, 17, 19, 20, 25, 28] described breastfeeding peer support offered to mothers with infants born with clinical illness or admitted with illness. These were categorised as unhealthy infants. In three studies [17, 19, 25], the infants were admitted in the NICU or the general paediatric ward with clinical conditions such as overweight [15], premature [20], diarrhoea [28] and malnutrition [13]. Five studies described breastfeeding peer support offered to both healthy and unhealthy infants while the remaining two studies were unclear on the status of the mother and/or infant being supported.

Second, the economic status of the mother was also a factor for eligibility to breastfeeding peer support programs. In six of the 18 studies [17, 19, 20, 25, 28] breastfeeding peer support targeted mothers from low income settings such as Latina or black women in the United States. In one study [18], breastfeeding peer support was targeted to only obese and overweight mothers who had given birth to overweight infants.

### Eligibility criteria for breastfeeding peer supporter role

The term breastfeeding peer supporter was defined in most of the articles based on required experience for the specific roles to be carried out by the individual. This definition varied slightly among the papers reviewed, with a majority of studies (15/18) defining a breastfeeding peer supporter as a mother who had successfully breastfed her own child for not less than six months or a mother who is still breastfeeding [13, 17–20, 22–25, 27–29, 31, 32]. Other eligibility characteristics included the breastfeeding peer supporter coming from the same community or locality as the mothers admitted in the index hospital [17–20, 26]. Additional inherent qualities for the BFPS included motivation or interest to offer breastfeeding support to other mothers [17, 27, 29, 31] and experience on specific conditions, for example, BFPS within NICUs have to be mothers who themselves had previously been admitted with their own child to a NICU [22–24]. Four studies required BFPS to have previously been trained on breastfeeding counselling [20, 21, 29, 32]. In one study [19], BFPS were simply described as mothers who have completed high school education.

### Mode of delivery of breastfeeding peer support

The articles indicated different modes of engagement for the BFPS at the hospital level as either employed, volunteers or subcontracted on different levels of remuneration (unpaid versus paid). The main mode of engagement was the employment which was described in 10 of the 18 articles and was facilitated through funding of research studies, county governments or directly to the hospitals. In three of the reviewed studies, BFPS were employed by the hospital through small grants or other sources of funding [22, 24, 26]. The county government [30, 33] and the public health department [32] also employed BFPS. In other studies, trained [27, 32] or untrained breastfeeding peer supporters volunteered their service [23, 27, 32]. In one study, through a partnership program, the breastfeeding peer supporters were trained and subcontracted to work in a hospital by an external institution. Employment within research institutions was done in the four of five randomized controlled trials [17, 18, 20, 21], and a pilot study [13]. Only two studies indicated the recruitment procedures for the BFPS: by placing an advertisement in the hospital job listing, word of mouth and contact with clients who had successfully breastfed their children. A prospective cohort pilot study in Kenya [13] initiated the recruitment process also through a job advertisement in the hospital’s noticeboard.

### Training of breastfeeding peer supporters

The majority (13) of the studies reported that the breastfeeding peer supporters had received some form of training before they were allowed into the hospitals. In these articles, training of breastfeeding peer supporters was developed using materials from a range of breastfeeding manuals [13, 17–22, 24, 26–29, 31] with no standardization across the studies and with differences in training content and duration. For example, in the 12 studies that described the length of training, the time it took to train a breastfeeding peer supporter ranged from 30 h [18] of classroom training to 10 weeks of training [27, 31] by a certified institution. Five studies had unclear description of training offered to the BFPS [25, 26, 30, 32, 33] and in one study it was reported that no breastfeeding support training was offered to the breastfeeding peer supporters [23].

In studies that had breastfeeding peer supporter training, introductory courses to breastfeeding and breastfeeding support was taught. In four studies, extensive training was offered on anatomy and physiology of breast and breastfeeding and how breastfeeding works [13, 17–19, 22]. Training on counselling skills was provided in five studies, while others were trained on the benefits of breastfeeding (*n* = 2) [13, 22], breastfeeding techniques (*n* = 2) [13, 20] and common breastfeeding difficulties [13]. Some breastfeeding programs were specifically targeted to infants who were admitted to hospital due to an illness. In such instances, training offered to BFPS was tailored to identify and manage neonatal danger signs and neonatal unit procedures [20], management of diarrhoea, anthropometric measurements of an infant with diarrhoea [21] and management of severe acute malnutrition for infants below six months [13]. In three studies, training was accompanied with role plays and hands-on experience [17, 18, 21] while in other studies training was followed by close supervision and assistance by a certified health professional [13, 17–19, 21].

### Roles of breastfeeding peer supporters in a hospital setting

In the articles, BFPS approached the mothers using different avenues including one-to-one or individualized, group support or a combination of the two. One-to-one support was mainly provided to mothers (*n* = 11) while one study used group support only [23]. One study used a combination of one-to-one support and group support [24], one applied either one to one support, group support or telephone support [26] and the practical guideline [29] recommended the use of either group support or one-to-one support. In (*n* = 11) articles, breastfeeding peer support was extended to mothers after hospitalization. The post-discharge follow-ups were done through home visits, telephone support and linkage to a community support group targeted at specific mothers who had received breastfeeding peer support at the hospital.

A description of the roles of BFPS was provided in most of the articles and included sharing breastfeeding knowledge and skills, managing breast problems, advice on proper nutrition and hygiene and providing emotional support. In eight studies, support was provided to improve mothers’ breastfeeding technique such as showing mothers how to properly position and latch the baby onto the breast [13, 17–19, 21, 26, 30, 32]*.* During counselling, BFPS explained to mothers the benefits of breastfeeding (*n* = 4) [17, 21, 29, 33], assisted mothers to understand feeding cues and breastfeeding frequency to achieve the right amount of feeds (*n* = 4) [13, 17–19], discouraged the use of bottles and pacifiers and behaviours that impede early initiation of breastfeeding [17] and discouraged mixed feeding [17, 21]. Other roles were the management of breastfeeding problems for example breast engorgement and inverted nipples [19] and also advising mothers on proper nutrition during breastfeeding [21, 22]. Breastfeeding peer supporters working in the NICU [24, 26] demonstrated to mothers the use and maintenance of breast pumps, milk storage techniques and assisted mothers to do Kangaroo Mother Care. They also participated in discharge planning to encourage breastfeeding post-discharge [24]. This was done through participation in weekly discharge planning rounds and identification of women who would benefit from post-discharge breastfeeding management then linking them with a community based lactation professional. The breastfeeding peer supporters also provided guidance on hand expression [32, 33], safe bottle feeding, breastfeeding assessment and identifying challenges to breastfeeding [13]. Breastfeeding peer supporters also performed the role of raising red flags and referral to the nutrition consultant (*n* = 3) [13, 26, 31] and also as a source of information and referral to community support [27, 30]. Elsewhere, they provided emotional support to mothers [22, 23, 25–27, 31, 33] (*n* = 7) by using their personal experiences to relate to and encourage mothers to be open minded and persist in following instructions. In three of the articles, BFPS acted as role models to the mothers they supported whereby they shared personal breastfeeding experiences.

### Breastfeeding peer supporters’ supervision

Supervision of peer supporters was described in most articles (*n* = 14) however, the professional role of the supervisors and frequency and mechanism varied. Supervision meant the peer supporter worked under guidance of a professional in the hospital unit. A lactation consultant was a supervisor in some articles [17, 18, 20, 22, 27] while others mentioned lactation program coordinator [24, 27, 28, 30], project coordinator [19], principal investigator of studies [21], infant feeding coordinator [31], breastfeeding network coordinator [33], and a paediatric nutritionist [13]. One practical guideline recommended that peer support supervision should be done by the trainer who offered peer support training as either individualized or group. In one study it was not clear who provided the supervision for the peer supporters [32] while in another, it was reported that no supervision was provided [23]. The mechanism and frequency of supervision on the other hand were described in two studies [13, 19]: biweekly meeting with the program coordinator to review cases and monthly one-hour continuous education [19] and working closely with the paediatric nutritionist to review a lactation plan [13].

### Evaluation of breastfeeding peer support programs

Breastfeeding peer support programs were evaluated in 13 of the 18 articles. Evaluation designs were either impact evaluation (*n* = 3), impact and process evaluation (*n* = 5) or process evaluation only (*n* = 5).

#### Impact evaluation

The impact evaluation was done using qualitative methods only (*n* = 4) [23, 24, 26, 27], quantitative only (*n* = 2) [22, 28] and mixed methods (*n* = 7) [13, 17–21, 25] evaluation designs. Quantitative evaluation assessed the impact of the breastfeeding peer support to increase the prevalence of exclusive breastfeeding as well as other breastfeeding outcomes such as initiation to breastfeeding [19], any breastfeeding [20, 22], or exclusive breastfeeding (*n* = 6) [18, 21, 25, 30, 32, 33] among the intervention group. Other studies evaluated the association between peer counselling and breastfeeding duration [28], meeting WHO breastfeeding discharge criteria for malnourished infants [13] and infant growth, morbidity and mortality [13].

#### Process evaluation

Five of the studies evaluated collected data on perceptions towards breastfeeding peer support. Breastfeeding peer supporters were perceived positively by health workers they interacted with [13, 24, 26, 27] and were described as insightful by coming up with useful tips on how to overcome breastfeeding challenges. Additionally, BFPS were acknowledged for playing a role other than breastfeeding support, for example helping mothers to easily adapt and cope with the hospital environment. In a study done in England [27] health workers viewed BFPS as “*singing from the same hymn*” after both health workers and BFPS received similar training*.* However, in one study health workers had friction with BFPS claiming that on some occasions breastfeeding peer supporters overstepped their boundaries [24] for example offering support in a case of spousal abuse which is the role of a social worker.

Four studies did an evaluation on the perception of mothers towards breastfeeding peer supporters. BFPS were perceived by mothers to be a source of encouragement and comfort (*n* = 3) [25, 26, 28]. Mothers reported that they were always there and found it easy to confide their fears in peer supporters. Mothers viewed the peer supporters as a source of practical support [13] to enable them to navigate through the confusing and frightening hospital environment. For example BFPS would be available to hold the infant for the mother to go and take a bath [13]. However, one study identified that information offered by BFPS and health workers could be inconsistent such as exclusive feeding practices in one instance [25]. In another study, mothers noted that there was insufficient information on the amount of milk mothers would have to express after discharge [13, 15].

Three studies evaluated the experience of the breastfeeding peer supporters themselves [13, 27, 28]. BFPS felt that the position was a motivation for them to get into a medical career like midwifery [27]. They cited the important role of good communication skills for supporting mothers and relation with the health workers [13]. Transitioning to being hospital staff was challenging for some BFPS, they found it difficult to relate with doctors as their colleagues especially when they were recently their doctors [26].

Of the 13 studies which were evaluated, the majority (11/13) indicated positive outcomes associated with BFPS, however two reported mixed observations. In one study, peer supporters were reported to not have impacted exclusive breastfeeding rates but increased the rate of any breastfeeding at discharge [18]. While in another study, peer supporters alone were reported to not improve breastfeeding outcomes unless paired with lactation consultants [22]. Overall breastfeeding peer supporters were successful in improving the prevalence of breastfeeding, early initiation of breastfeeding and retention of exclusive breastfeeding. Additionally, malnourished infants’ u6m attained WHO breastfeeding discharge criteria through breastfeeding peer support [13], control of diarrhoea and the BFPS developed positive relationships with other hospital staff and mothers [21].

## Discussion

Our review included 18 articles describing inpatient breastfeeding peer support strategies, whereby 13 were publications from peer reviewed journals. Our results indicate that breastfeeding peer supporters have been used in different hospital settings to support breastfeeding of both healthy and unhealthy infants. However, most of the articles found have reported breastfeeding peer support in high income settings such as the United States and Europe. Only two study reports were from LMICs.

Positive outcomes were reported in 13 articles which conducted evaluation for the breastfeeding peer support programmes. Outcomes assessed included prevalence of breastfeeding, early initiation and exclusive breastfeeding rates. Primary data from quantitative evaluation of the nine peer reviewed publications suggest that breastfeeding peer supporters are effective to increase early initiation of breastfeeding, rates of breastfeeding continuation and re-establishing exclusive breastfeeding among hospitalized ill-malnourished infants. There are several reasons that underpin the reported success of in-patient breastfeeding peer support strategies. A review by McFadden et al., 2017 [34] concluded that effective breastfeeding interventions will have the following key principles: firstly, the intervention will offer timely (within mother’s schedule) one-to-one support to mothers, secondly support will be offered more proactively and thirdly support will be offered continuously over a period of time (five or more contact points) [35]. From our review, within an in-patient setting, breastfeeding peer supporters have the opportunity to maintain daily one-on-one contact with mothers of admitted infants over a continued period of time (a few days) [13, 17–19, 22, 28] and opportunities to extend the contact into post-discharge follow-up period. In this way, in-patient breastfeeding peer support strategies meet the key principles for successful breastfeeding interventions.

Even though breastfeeding peer support strategies are not new to the health system, we found that in an in-patient environment the eligibility criteria, identification, training and supervision of breastfeeding peer supporters differs from one context to another. Similar to other peer support programs, BFPS were described with the concepts of shared experiences and social matching. In our review, consistently, BFPS were defined as mothers often from a similar community, as mothers of admitted infants who have current or previous breastfeeding experience and are interested in supporting others who may be facing breastfeeding challenges. Other important concepts were age of the BFPS to be similar with most mothers, they should also have similar culture and heritage and in some instances education level was important. To adopt breastfeeding peer support strategies into the health system a consistent definition of peer supporters is important as it will help guide objective identification and recruitment and facilitate comparability of results from different in-patient peer support interventions. In addition to shared experience and social matching, our results highlight another important peer support characteristic, the ability to provide emotional, appraisal and informational support. This finding is consistent with what has been described in a content analysis by Dennis et al. [36] and suggests that shared experience, social matching and an additional inherent skill to provide mothers with emotional and informational support are the key qualities of a good peer supporter. This is important as identifying the right peer supporter is a crucial step towards a successful breastfeeding support programme.

Ideally, a clearly defined role should result in a well-defined and standardized training package. Findings from our review indicate that in most studies although the breastfeeding peer supporters’ roles were clearly defined, this did not always translate to an equally well-organised and well-thought-out training package. The training described in the reports were not consistent in content or duration. Where training was offered the training content and materials differed and mostly was custom made from existing UNICEF, WHO and national breastfeeding manuals [9, 37–39]. In contrast, community based breastfeeding peer support programmes, including the baby-friendly community initiative, have well-developed, well-structured and well-documented training manuals and implementation tools which has enabled standardization and evaluation of the community support program [10]. Training for peer supporters should avoid creation of paraprofessionals who might not only take on roles of other healthcare workers but also diminish the intended peer commonality with the clients [36]. The aim of standardization is to develop a manual that can be replicated in different settings based on need and it will also facilitate future comparability of outcomes. Training of peer supporters is essential in provision of quality breastfeeding support, hence using consistent training packages is key to facilitate uptake of in-patient breastfeeding strategies in LMIC.

Like other health workers of similar cadre, such as community health workers, we found that a variety of mechanisms are applied to engage them. In our review, the majority of the BFPS were engaged through enumerated employment. A few reports described in-patient support offered by volunteers [23, 27, 31]. Compared to volunteers, engaging peer supporters in a formal enumerated employment arrangement provides several advantages including the fact that it makes it easier to standardize the recruitment, training and management process. According to an article by Cherrington et al. volunteers were reported to demand respect for their time and wanting to work within their own schedule making it difficult to standardize recruitment, management and evaluation of their performance [40]. Our findings are in agreement with findings from studies that have evaluated the role of community health workers and reported that employed workers’ activities were organised making it easy to manage within fixed schedules and specified roles [40]. Our findings seem to suggest that in-patient peer support interventions could consider engaging peer supporters as enumerated employees as this would ensure implementation of defined roles with assured availability over time. Employment would also simplify their recruitment, management and evaluation of breastfeeding peer support strategies.

Supervision is a key component of any peer support intervention. Our review reveals that within the hospital setting, peer supporters do not work unsupervised. BFPS in both HIC and LMIC settings were supervised by a health professional in the hospital. Supervision offered by senior health workers within the in-patient setting also worked to swiftly integrate breastfeeding peer supporters into the health system and provide monitoring of activities which is key to maintenance of professionalism by the peer supporters and leads to acceptability of their role by other health workers. This supervision approach is similar to that offered to other health workers of a similar cadre such as community based peer supporters and community health workers [41]. In addition to direct supervision, during implementation providing written guidelines and Standard Operating procedures was reported to underpin a structured and well documented breastfeeding support process by peer supporters [13, 20]. Structured supervision and documentation are essential to ensure accountability and provide a point of reference for breastfeeding support offered to mothers within a hospital setting. Documentation becomes a source of information to other health workers and an important tool for providing continuity of care where handing over is required. Documentation is also important in evaluating workload for the peer supporters and conducting objective performance reviews. Our findings therefore further emphasised the importance of consistent documentation and supervision processes to breastfeeding peer support interventions in all contexts.

### Limitations

The literature included in this review were mainly from the United States of America and the United Kingdom, where breastfeeding peer support has been integrated into the health system for example through the La Leche League international. Such considerations influence the interpretation of findings especially for the LMIC contexts since the majority of results are from HICs. Second, the research project was undertaken in fulfilment of a post-graduate diploma and hence had resource limitations that constrained an extensive handsearching for grey literature.

## Conclusions

Breastfeeding peer support strategies are applied in different hospital settings and can be used to improve breastfeeding outcomes of ill hospitalised u6m. However, to achieve integration, scalability and comparability of impact and outcomes, there is a need to standardize training and to develop consistent implementation and supervision plans of in-patient peer supporters’ strategies across different settings. Further research to assess sustainability and evaluate cost-effectiveness of in-patient breastfeeding peer support strategies will improve uptake and scalability of these potentially lifesaving interventions.

## Data Availability

The datasets used and/or analysed during the current study are available with this document.

## Abbreviations

BFPS: Breastfeeding peer supporters
HIC: High income countries
LMIC: Low and Middle-income countries
MOH: Ministry of Health
u6m: Under 6 months
WHO: World Health Organization
